# CD24 is an independent prognostic marker of survival in nonsmall cell lung cancer patients

**DOI:** 10.1038/sj.bjc.6600702

**Published:** 2003-01-28

**Authors:** G Kristiansen, K Schlüns, Y Yongwei, C Denkert, M Dietel, I Petersen

**Affiliations:** 1 Institute of Pathology, Charité University Hospital, Schumannstr. 20/21, 10117 Berlin, Germany

**Keywords:** nonsmall cell lung cancer, CD24, tissue microarray, immunohistochemistry

## Abstract

Originally identified as a B-cell marker, expression of the cell surface molecule CD24 has meanwhile been observed in a variety of human malignancies. It appears to function as a ligand of P-Selectin, an adhesion molecule that is present in activated platelets and endothelial cells. We aimed to determine the rate of CD24 expression in our nonsmall cell lung cancer (NSCLC) collection and to clarify its correlation with clinicopathological parameters including patients' survival. A total of 89 NSCLC were analysed immunohistochemically using a monoclonal CD24 antibody (clone 24C02) and a standard detection system (LSAB, DAKO) on NSCLC tissue microarrays (TMA). The staining was semiquantitatively scored (0, 1+, 2+, 3+) and grouped into high (2+, 3+)- and low (0, 1+)-level expression for statistical analysis. A high level of CD24 expression was observed in 45% of the cases, preferentially adenocarcinomas. Patients whose tumours had a high CD24 expression showed a significantly shorter median survival time of 23 months *vs* 38 months (*P*=0.033, log-rank test). Similarly tumour, grading, nodal status and clinical stage were significant prognostic markers in univariate survival analysis. Importantly, in the Cox regression-based multivariate analysis, CD24 expression (*P*=0.025) together with tumour stage (*P*=0.006) and grade (*P*=0.011) proved to be independent prognostic parameters. We hypothesise that the decreased survival of NSCLC patients with strongly CD24-positive tumours is related to an enhanced propensity of haematogenous metastasis formation, which might be P-Selectin mediated.

Lung cancer is a major cause of death from neoplastic malignancy in the Western world. In the USA alone, 169 400 new cases are expected for the year 2002 with 154 900 expected deaths from this disease (Jemal *et al*, 2002). In the last 20 years, the mortality rate could be lowered only by 6% because of improved therapies. So far, the most promising approach appears to be primary and secondary prevention (Henschke *et al*, 1999).

The molecular biology of nonsmall cell lung cancer (NSCLC), the largest lung cancer subgroup, is under intense international investigation in order to characterise new molecular marker genes which might be helpful in diagnosis or therapy. A gene that recently has gained new interest is CD24. It is a small heavily glycosylated mucin-like glycosyl-phosphatidylinositol(GPI)-linked cell surface protein (Pirruccello and Le Bien, 1986; Fischer *et al*, 1990; Akashi *et al*, 1994), which is physiologically expressed not only in developing (Shirasawa *et al*, 1993; Poncet *et al*, 1996; Cram *et al*, 1999) or regenerating (Figarella-Branger *et al*, 1993) tissue, but also in granulocytes, pre-B-cells, keratinocytes (Redondo *et al*, 1998) and renal tubules (Droz *et al*, 1990). In neoplasia, CD24 expression has been described not only in haematologic malignancies (Pirruccello and Lang, 1990; Raife *et al*, 1994; Lavabre-Bertrand *et al*, 1994), but also in a large variety of solid tumours, for example, renal cell carcinoma (Droz *et al*, 1990), small cell lung cancer (Jackson *et al*, 1992), nasopharyngeal carcinoma (Karran *et al*, 1995), hepatocellular carcinoma (Huang and Hsu, 1995), bladder carcinoma (Gromova *et al*, 1999), glioma (Senner *et al*, 1999), breast cancer (Fogel *et al*, 1999; Yang *et al*, 1999; Liu and Vadgama, 2000) and ovarian cancer (Welsh *et al*, 2001; Kristiansen *et al*, 2002). CD24 is a ligand of P-selectin (Sammar *et al*, 1994), an adhesion receptor on activated endothelial cells and platelets, and could thus contribute to the metastasising capacities of CD24-expressing tumour cells (Aigner *et al*, 1995,^1997^,^1998^; Friederichs *et al*, 2000). Recently, we described CD24 as an independent prognostic marker of shortened patient survival in ovarian cancer (Kristiansen *et al*, 2002). To date very little is known about CD24 in NSCLC.

The aim of this study was to evaluate the status of CD24 expresssion in our set of NSCLC patients and to investigate the association of CD24-protein expression determined by immunohistochemistry with clinicopathological parameters, disease stage according to UICC and patient survival.

## MATERIALS AND METHODS

### Tumour samples

Our collective consisted of 89 patients between age 34 and 80 years (mean 62) who underwent thoracotomy for resection of NSCLC in the Department of Surgery at the Charité University Hospital from 1995 to 1997, and whose clinical follow-up data were available. No adjuvant radiotherapy or chemotherapy was applied before surgery. Table 1

**Table 1:** Relationship between CD24 expression and various clinicopathological parameters

**Characteristics**	**Sum**	**CD24 low**	**CD24 high**	***P*-value**
Total no.	89	49	40	
				
Adeno	49	19	30	**0.001**
SCC	40	30	10	
				
G1/G2^a^	47	26	21	1
G3	42	23	19	
				
pT1	22	12	10	1
pT2+ b	67	37	30	
				
pN0	53	33	20	0.129
pN1+ c	36	16	20	
				
Stage I–II^d^	62	36	26	0.488
Stage III^d^	27	13	14	

a G1 (*n*=3), G2 (*n*=44).

b pT2 (*n*=57), pT3 (*n*=8), pT4 (*n*=2).

c pN1 (*n*=12), pN2 (*n*=23), pN3 (*n*=1).

d Stages I (*n*=49), II (*n*=13), III (*n*=27).

summarises the clinicopathological characteristics according to TNM criteria of the UICC (Sobin and Wittekind, 1997). The histopathological diagnosis was established in every case according to the WHO guidelines (Travis, 1999). Only primary carcinomas were included in the study.

### Tissue array generation

A tissue microarray (TMA) was constructed as previously described (Kristiansen *et al*, 2001). Briefly, suitable areas for tissue retrieval were marked on standard haematoxylin–eosin (HE) sections, punched out of the paraffin block and inserted into a recipient block. The tissue arrayer was purchased from Beecher Instruments (Woodland, USA). The punch diameter was 0.6 mm. The lung tumour array consisted of 127 tissue samples: 89 tumours and 38 punches of normal or peritumoural lung tissue. For comparison of the tissue array immunostaining with conventional slides, whole mount paraffin sections were cut from 15 cases that were incorporated in the tissue array.

### Cell lines and primary cell culture

Four primary tumour cell lines of lung adenocarcinomas were established in our laboratory derived from patients with primary cancers who had surgery for NSCLC at the Charité University Hospital (D51, D54, D97, D117). These cells were grown in Leibovitz 15 media supplemented with 10% FCS and 1% glutamine. Small cell lung cancer cell lines (H526, H378, DMS-79, SHP-77) and the lung squamous cell cancer cell line H2170 were purchased from the ATCC (Rockville, MD, USA). Lung adenocarcinoma cell lines A427 and A549 were purchased from the German Collection of Microorganisms and Cell Cultures (Braunschweig, Germany). All cells were grown as recommended. The cell lines were centrifuged and the pellets were formalin fixed and embedded in 0.8% agarose. These agarose pellets were finally paraffinised and conventionally sectioned for immunohistochemistry.

### Immunohistochemistry

The tissue array block was freshly cut (4 *μ*m), sections were mounted on superfrost slides (Menzel-Gläser, Germany) and dewaxed with xylene, and gradually hydrated. Antigen retrieval was achieved by pressure cooking in 0.01 M citrate buffer for 5 min. The primary CD24-antibody (Ab-2, clone 24C02, Neomarkers, USA) was diluted 1 : 100 using a background reducing dilution buffer from DAKO (Germany). No other blocking agents were employed. The primary antibody was incubated at room temperature for 1 h. Detection took place by the conventional labelled-streptavidin–biotin (LSAB-kit, DAKO, Germany) method with alkaline phosphatase as the reporting enzyme according to the manufacturer's instructions. Fast-Red (Sigma-chemicals, Hamburg) served as chromogen, afterwards the slides were briefly counterstained with haematoxylin and aquaeously mounted.

The CD24 staining was independently examined by two clinical pathologists (GK, YY). The staining intensity was semiquantitatively scored from absent to strong (0, 1+, 2+, 3+) for each case. Two TMA were processed to reduce case dropout.

### Statistical analysis

Fishers exact test, which is especially suited for small sets of data, was used to determine the strength of association between the investigated parameters. To compare the expression of CD24 with clinicopathological parameters, 2×2 contingency tables (e.g. CD24 score 0–1 *vs* 2–3 and G1& G2 *vs* G3) were set up and the *P*-values were calculated. Cumulative survival curves were calculated according to the Kaplan–Meier method, differences in survival time were assessed with the log-rank test. Multivariate survival analysis was performed on all parameters that were found to be significant on univariate analysis using the Cox regression model. *P*-values <0.05 were considered significant. All calculations were performed using the statistical software package SPSS, version 10.0.

## RESULTS

### Immunohistochemistry

**Figure 1:**
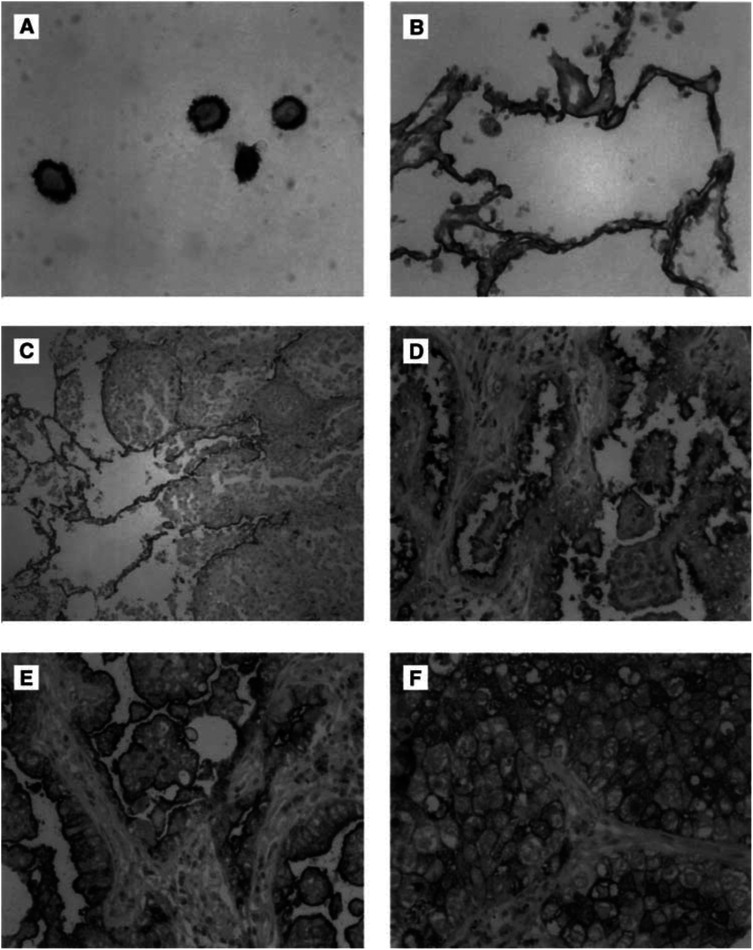
CD24 immunohistochemistry (**A**–**F**) in NSCLC. (**A**) Adenocarcinoma cell line with strong membraneously accentuated CD24 expression. (**B**) Normal lung parenchyma: membraneous expression in pneumocytes. (**C**) Normal lung parenchyma (on the left) with infiltrates of a poorly differentiated adenocarcinoma without CD24 expression (on the right). (**D**) Adenocarcinoma with moderate CD24 expression (2+). (**E**) Adenocarcinoma and (**F**) Squamous cell carcinoma with high CD24 expression (3+).

Of the 11 lung cancer cell lines, we observed a strong membraneous CD24 expression in the adenocarcinoma cell line D51 (Figure 1A

). Four cell lines had a cytoplasmic staining, which was less pronounced (A427: ADC cell line; H378, DSM-79: small cell lung cancer cell lines; H2170: SCC cell line). Six cell lines were negative.

Normal lung parenchyma showed a strong membraneous staining pattern of pneumocytes (Figure 1B), whereas bronchial epithelium was mostly negative. In tumour tissue of primary lung carcinomas, CD24 immunoreactivity was observed in 68 (76%) cases with a cytoplasmic and membraneous staining pattern. The gross slides generally showed a homogenous staining of the tumours, which is a necessary condition to obtain reliable data from small tissue array punches. Specifically, on direct comparison of intensity grades of conventional whole mount sections and arrayed tissue of the same case (*n*=15), we found eight identical gradings, six cases were slightly different, but still fell into the same group (0–1=low-level expression *vs* 2–3=high-level expression), only a single case was discordant. This demonstrates that the CD24 staining of the tissue punch is representative (*P*=0.001, Fishers exact test).

The TMAs allowed for evaluation of 89 cases. In all, 21 cases (24%) exhibited no relevant CD24 staining (Figure 1C). In 28 cases (31%), a minimal staining was observed. A total of 21 cases (24%) showed a moderate staining intensity (Figure 1D) and 19 cases (21%) had a strong staining signal (Figure 1E, F). Taking groups three and four together, 45% of tumours showed a high level of expression of CD24 in this study ( Table 1). This was opposed to no or minimal CD24 expression (0, 1+) in the statistical analysis.

### Statistical analysis

CD24 expression was significantly higher in adenocarcinomas ( Table 1, Fishers exact test, *P*=0.001). No correlation was found with tumour grading (*P*=1), tumour size (*P*=1), nodal status (*P*=0.129) or disease stage according to UICC (*P*=0.488).

#### Univariate survival analysis

Cumulative survival curves were calculated according to the Kaplan–Meier method, differences in survival were assessed with the log-rank test (Table 2

**Table 2:** Univariate survival analysis (Kaplan–Meier)

**Characteristic**	**No. of cases**	**Mean survival time (months±s.e.)**	**Median survival time (months±s.e.)**	***P*-value**
Histology				
Adeno	49	39±4	36±10	0.566
SCC	40	32±3	30±4	
Grading				
G1&2	47	45±4	46	**0.001**
G3	42	24±3	19±3#	
Tumour size (pT)				
pT1	22	47±5	n.r.^a^	0.083
pT2+	67	32±3	30±4	
Nodal status				
PN0	53	45±4	n.r.	**0.0001**
PN1+	36	22±3	18±3	
Stage				
I–II	62	44±3	46	**0.0005**
III	27	21±3	17±5	
CD24 expression				
Low	49	40±3	38	**0.033**
High	40	31±4	23±3	

a n.r.=not reached.

). First, we analysed conventional and well-described clinicopathological parameters. Kaplan–Meier curves demonstrated a significant impact of tumour grading (*P*=0.001), nodal status (*P*=0.0001) and tumour stage (*P*=0.0005) on patient survival (Figures 2A–C

**Figure 2:**
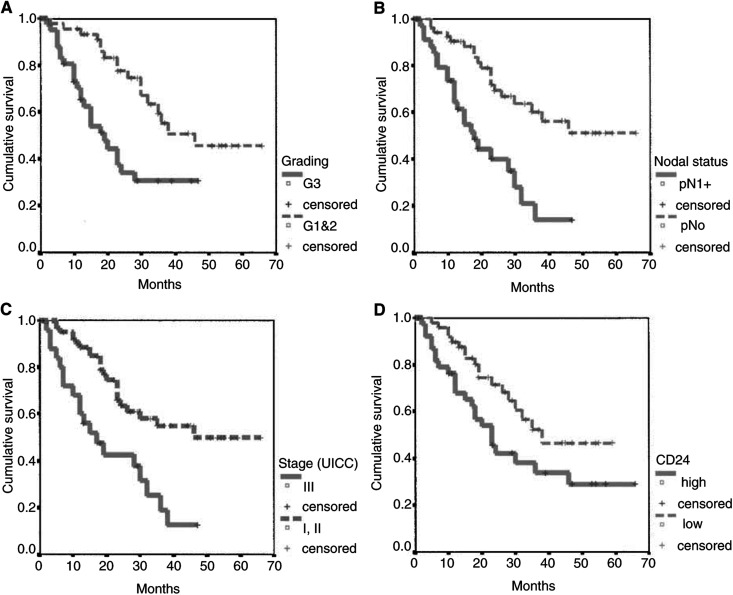
Survival analysis. Kaplan–Meier curves (**A**–**D**): (**A**) tumour grading (*P*=0.001); (**B**) nodal status (*P*=0.0001); (**C**) disease stage (*P*=0.0005); (**D**) CD24 expression (*P*=0.033).

). Tumour size (pT1 *vs* pT2+) was of borderline significance (*P*=0.083). Histological tumour type did not show a significant impact on patient survival time (*P*=0.566), although the mean and median survival times of squamous cell carcinomas were shorter.

For CD24 expression, we found a statistically significant difference of median survival time for tumours with low levels (0, 1+) compared to tumours with high levels (2+, 3+) of CD24 expression (Figure 2D) with 23 months *vs* 38 months (*P*=0.033).

#### Multivariate survival analysis

A multivariate progression analysis based on the Cox proportional hazard model was performed to test the independent value of each parameter predicting overall survival (Table 3

**Table 3:** Multivariate survival analysis (Cox regression model)

	**Beta**	**Standard error**	**Wald**	**df**	**Relative risk (RR)**	**95% CI of RR**	***P*-value**
Grading	0.827	0.327	6.411	1	2.286	1.205–4.336	0.011
Stage (UICC)	0.877	0.320	7.491	1	2.404	1.283–4.505	0.006
CD24	0.708	0.313	5.122	1	2.030	1.100–3.750	0.024

). In this analysis, we included tumour grading (G1 and G2 *vs* G3), disease stage (I–II *vs* III–IV) and CD24 expression (0, 1+ *vs* 2+, 3+). All three parameters, tumour grading (*P*=0.011), disease stage (*P*=0.006) and CD24 expression (*P*=0.024) proved to be independent prognostic factors for shortened overall survival in NSCLC.

## DISCUSSION

In this study, 11 lung cancer cell lines and tissue of 89 NSCLC cases were immunohistologically examined for the expression of CD24 protein using a well-characterised monoclonal antibody. We found CD24 expression in 45% (five out of 11) of the cell lines: two adenocarcinoma cell lines (two out of six), two small cell lung cancer cell lines (two out of four) and one squamous cell cancer cell line (one out of one). This appears to match the data of Jackson *et al*, who reported CD24 expression in SCLC cell lines and in some adenocarcinoma cell lines, although unfortunately no details were given for the NSCLC cell lines investigated (Jackson *et al*, 1992). We found a higher incidence of CD24 expression (76% of cases) in NSCLC tissue. These different incidences of CD24 expression in cell lines and tissues might be explained in several ways. First, CD24-positive NSCLCs might be more difficult to culture as cell lines than CD24-negative tumours. Second, CD24 staining in tissue sections was often accentuated at cell–cell contacts, which are mostly missing in cell lines.

To our knowledge, this is the first study to examine the CD24 expression in a larger tumour sample of NSCLC. Methodologically, the usage of TMAs was very convenient to help processing a large set of cases. The validity of this approach was confirmed by comparison of the CD24 array staining results with matching conventional whole mount sections.

We found CD24 expression to be an independent predictor of shortened patient survival as evidenced by univariate and multivariate analyses in NSCLC. This raises the question of the biological function of CD24 in NSCLC. CD24 is expressed in a large variety of human tissues: physiologically, in developing or regenerating tissues (Figarella-Branger *et al*, 1993; Shirasawa *et al*, 1993; Poncet *et al*, 1996; Cram *et al*, 1999) and a few mature cell types such as, for example, granulocytes, keratinocytes (Redondo *et al*, 1998) and renal tubules (Droz *et al*, 1990). Pathologically, CD24 has been described in B-cell neoplasia (Pirucello and Lang, 1990; Raife *et al*, 1994; Lavabre-Bertrand *et al*, 1994), renal cell carcinoma (Droz *et al*, 1990), small cell lung cancer (Jackson *et al*, 1992), nasopharyngeal carcinoma (Karran *et al*, 1995), hepatocellular carcinoma (Huang and Hsu, 1995), bladder carcinoma (Gromova *et al*, 1999), glioma (Senner *et al*, 1999), breast cancer (Fogel *et al*, 1999; Liu and Vadgama, 2000) and ovarian cancer (Welsh *et al*, 2001; Kristiansen *et al*, 2002). This ubiquitous expression obviously excludes a diagnostic use of CD24 as a specific marker for NSCLC or any other tumour type.

CD24 has been identified as a ligand to P-selectin (Sammar *et al*, 1994), and it is conceivable that this function contributes to a more aggressive metastatic behaviour of CD24-positive tumour cells, as *in vitro* evidence suggests (Aigner *et al*, 1995,^1997^,^1998^; Friederichs *et al*, 2000). P-selectin is a surface molecule expressed by activated endothelial cells and platelets (McEver, 1991). It plays an important role in marginal adhesion and migration of cells under shear forces in the blood stream. Moreover, P-selectin deficiency has been linked to decreased rates of metastasis formation in mice, which underscores the relevance of this adhesion receptor in tumor progression (Kim *et al*, 1998). Physiologically, its primary ligand is P-Selectin Glycoprotein Ligand-1 (PSGL-1), which is expressed, for example, by neutrophils. Possibly, CD24-expressing tumour cells can spread more easily because of their capacity to either form thrombi with activated platelets or to adhere to endothelia in the bloodstream as demonstrated for CD24-expressing breast cancer cells (Aigner *et al*, 1997). This mechanism of metastasis does not only require expression of CD24 of the tumour, but also expression of P-selectin in platelets and endothelia of the vasculature of the target organs. Obviously, P-selectin expression on platelets and endothelia of lung cancer patients can hardly be studied comprehensively *in vivo*. Importantly, P-selectin-mediated binding of SCLC to endothelial cells has already been shown *in vitro* (Pottratz *et al*, 1996) and the CD24/P-selectin pathway was found to initiate lung colonisation of the human lung adenocarcinoma cell line A125 in a mouse model (Friederichs *et al*, 2000).

These observations are consistent with the view that CD24 is a cell surface protein that might be important for haematogenous metastasis formation, and we feel that this is the most likely explanation of the decreased survival times of strongly CD24- expressing tumours because most lung cancer patients die from systemic metastatic disease. In our study, clinicopathological parameters were only considered at the time of surgery. Thus, we did not find an association of CD24 with clinical stage since patients with haematogenous metastases are excluded from surgical therapy and are consequently not represented in our tumour sample. Also, the nonsignificant association of CD24 expression with nodal status is compatible with this interpretation, since lymphatic spread is a biologically different process, although it frequently precedes haematogenous dissemination.

This study establishes CD24 expression as an independent prognostic tumour marker in NSCLC. Our recent description of CD24 expression as an independent marker of shortened patient survival in epithelial ovarian cancer (Kristiansen *et al*, 2002) further underscores the importance of CD24 in metastatic disease progression of human carcinomas. Prospectively, CD24 might aid the clinician in the selection of an appropriate therapy for individual patients. In this circumstance, it is of relevance to know that the intravenous administration of CD24-specific antibodies has been sucessfully used therapeutically to treat transplantation-associated B-cell proliferative syndrome (Fischer *et al*, 1991; Garnier *et al*, 2002). This new therapeutic option might deserve consideration in NSCLC patients as well.
